# Close Call From a Sweet Twist: A Case of Licorice-Induced Torsades De Pointes

**DOI:** 10.7759/cureus.34126

**Published:** 2023-01-24

**Authors:** Victor Molina-Lopez, Andrew Engel-Rodriguez, Jose Escabi-Mendoza

**Affiliations:** 1 Cardiovascular Disease, Veterans Affairs (VA) Caribbean Healthcare System, San Juan, PRI; 2 Internal Medicine, Veterans Affairs (VA) Caribbean Healthcare System, San Juan, PRI

**Keywords:** pseudohyperaldosteronism, qt prolongation, other causes of hypokalemia, supplements, secondary hypertension, refractory hypokalemia, torsades de pointes (tdp), hypokalemia, resistant hypertension, licorice

## Abstract

Torsades de pointes (TdP) is a life-threatening cardiac arrhythmia that can result from QT interval prolongation, sometimes secondary to medication adverse effects and electrolyte derangements. We present a 95-year-old Hispanic male with advanced chronic kidney disease (CKD) that was evaluated for dizziness and progressive weakness. The diagnosis of severe symptomatic hypokalemia and QT prolongation was made, and the patient was admitted for telemetry monitoring and aggressive intravenous electrolyte replacements. While under observation, the patient experienced syncope due to ventricular tachycardia (VT) with episodes of torsades de pointes. Due to refractory potassium depletion and hypertension, workup for hyperaldosteronism revealed renal potassium wasting, inappropriately normal plasma renin levels, and almost undetectable aldosterone levels. Careful analysis revealed the excessive chronic daily ingestion of licorice-containing candy twists and tea, which may cause pseudohyperaldosteronism. Licorice is a commonly used natural product that is available in many forms. It is sometimes used as a natural supplement and as a sweetener that can be widely found in many food products. Excessive ingestion can lead to apparent mineralocorticoid excess, reduced plasma potassium, sodium retention, hypertension, and metabolic alkalosis. Hypokalemia can be severe in some patients and lead to fatal cardiac arrhythmias such as ventricular tachycardia and torsades de pointes. Careful analysis is essential in cases of refractive hypokalemia and renal potassium wasting, especially in elderly patients with underlying renovascular disease.

## Introduction

Licorice is a perennial plant native to Asia Minor area and the Mediterranean. Triterpene saponins are the main constituents, of which glycyrrhizic acid is the main component [1]. The US Food and Drug Administration (FDA) recognizes licorice and its derivatives as safe; however, they have issued warnings about larger consumption in at-risk groups. Pseudohyperaldosteronism is one of its lesser-known side effects [2]. It is a clinical condition characterized by sodium retention, hypertension, metabolic alkalosis, potassium wasting, and suppression of plasma renin and aldosterone levels [2]. This can be due to prolonged intake of products flavored with licorice such as licorice roots, teas, tobacco, herbal products, candies, breath fresheners, and even laxatives [3]. Glycyrrhizic acid causes a cortisol-induced mineralocorticoid reaction and also has the capacity to directly stimulate the mineralocorticoid receptors (MR) [4]. Torsades de pointes (TdP) is a life-threatening cardiac arrhythmia that can result from disorders affecting myocardial repolarization, prolonging the QT interval. Acquired forms of long QT syndrome usually result from drug therapy, ischemic heart disease, and electrolyte disorders such as hypokalemia or hypomagnesemia [5]. This rhythm disorder may cause dizziness, syncope, and even sudden cardiac death. In patients with unclear etiology for hypokalemia, physicians should carefully explore medication use, including nutritional supplements and other food products. Licorice-containing products contain glycyrrhizin, which may have indirect life-threatening cardiac rhythm effects through its effect on potassium levels [3].

## Case presentation

**Figure 1:**
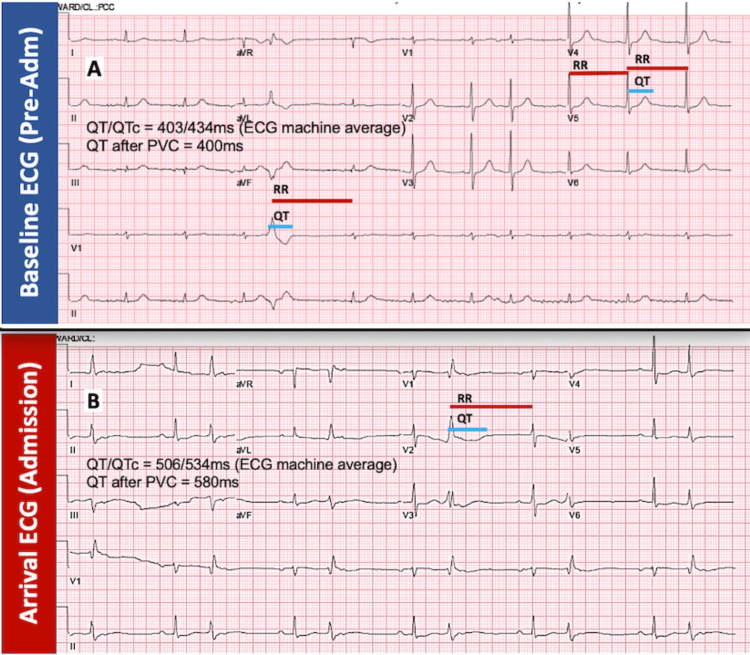
Baseline and arrival ECG (A) The patient’s baseline ECG without QT interval prolongation and with an averaged QT/QTc of 403/434 ms and post-premature ventricular contraction (PVC) QT of 400 ms. (B) Admission ECG with evidence of frequent premature ventricular contractions (PVCs) in bigeminy pattern, T-wave flattening, and prolonged QT intervals averaged at 534 ms and post-PVC at 580 ms Adm, admission; ECG, electrocardiogram; QTc, corrected QT

A 95-year-old Hispanic male with a past medical history of coronary artery disease with past coronary bypass surgery, heart failure with preserved ejection fraction, type 2 diabetes mellitus, chronic kidney disease (CKD) stage 4, anemia of chronic disease, and hypothyroidism visited the emergency department (ED) for the evaluation of a four-day history of general malaise and dizziness, which was associated with progressive weakness and decreased oral intake. He denied fever, chills, nausea, diarrhea, vomiting, smoking, diuretic use, recent illness, recreational drug use, or alcohol consumption. His family history was not significant. Vitals were remarkable for marked hypertension (temperature, 98°F; blood pressure, 184/102 mmHg; heart rate, 72 beats per minute {bpm}; respirations, 13 breaths per minute; and oxygen saturation, 97%). Chest auscultation was unremarkable, and the abdominal examination did not reveal tenderness, hepatomegaly, or vascular bruits. His arrival 12-lead electrocardiogram (ECG) in the ED (Figure 1B) revealed a sinus rhythm with a first-degree atrioventricular block, frequent premature ventricular contractions (PVCs) in a bigeminy pattern, T-wave flattening, and prolonged QT interval averaged at 534 milliseconds, more pronounced after PVC (580 ms), not present on prior reference ECG (Figure 1A). Medication reconciliation did not reveal any QT-prolonging medications. His chronic outpatient medications were clopidogrel 75 mg daily, levothyroxine 100 μg daily, metformin 1000 mg twice a day (bid), nifedipine 60 mg daily, hydralazine 25 mg bid, and chlorthalidone 25 mg daily. Initial laboratory data (Table 1) showed no leukocytosis, normal platelets, anemia within the patient’s baseline range, severe hypernatremia, severe hypokalemia, normal chloride, elevated bicarbonate (HCO_3_), mild azotemia, stable renal function at CKD stage 4 baseline range, normal calcium, hypomagnesemia, normal phosphate, and blood glucose within hospitalization goal. A chest X-ray did not reveal vascular congestion or pulmonary consolidations.

**Table 1:** Initial and specialized laboratory values with reference ranges

Laboratory test	Laboratory value	Laboratory range
	Initial laboratory results	
Hemoglobin	9.6 g/dL	12.6-17.8 g/dL
White blood cells	9.2 L × 10^3^/μL	4.3-9.3 L × 10^3^/μL
Platelets	185 L × 10^3^/μL	155-371 L × 10^3^/μL
Sodium	149 mEq/L	135-145 mEq/L
Potassium	2.3 mEq/L	3.5-5.0 mEq/L
Chloride	100 mEq/L	100-110 mEq/L
Bicarbonate	35 mEq/L	24-32 mEq/L
Blood urea nitrogen	35	10-26 mg/dL
Creatinine	2.5 mg/dL	0.7-1.5 mg/dL
Glucose	113	70-99 mg/dL
Calcium	9.0 mg/dL	8.5-10.5 mg/dL
Magnesium	1.7 mEq/L	1.8-2.4 md/dL
Albumin	4.1 g/dL	2.6-5.2 g/dL
	Specialized laboratory results	
Urine potassium	14 mEq/L	
Urine creatinine	63 mg/dL	
Urine potassium/urine creatinine ratio	22.2	>20 suggests potassium wasting
Plasma renin activity (PRA)	0.24 ng/mL/hour	0.25-5.82 ng/mL/hour
Plasma aldosterone concentration (PAC)	<1 ng/dL	3-16 ng/dL
PAC/PRA ratio	4.1	>30 suggests primary hyperaldosteronism

**Figure 2:**
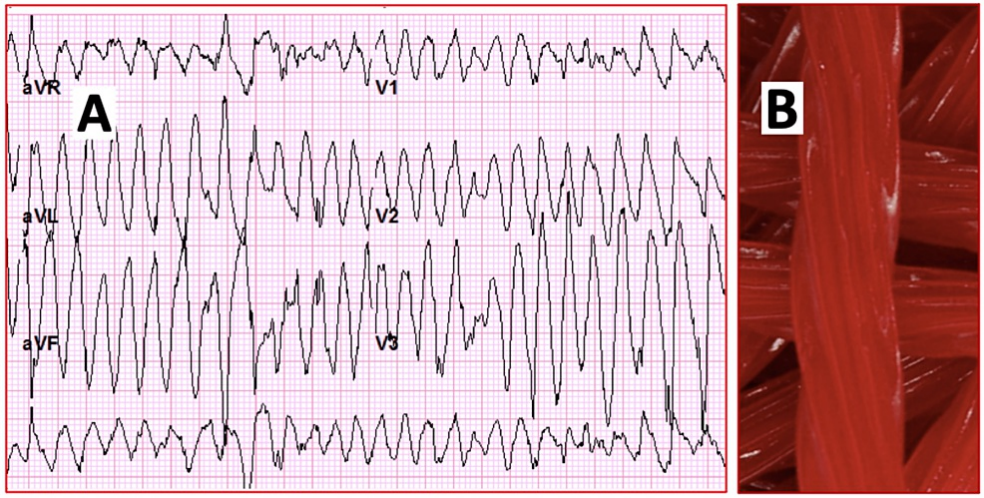
Torsades de pointes and licorice candy twists (A) ECG trace obtained during polymorphic ventricular tachycardia on the cardiac telemetry, with evidence of torsades de pointes (TdP) and the characteristic twisting of peaks. (B) The illustration shows chewy ropes of candy licorice twists as a common source of licorice and hence the metaphoric title of “Close Call From a Sweet Twist,” referring to the licorice-provoked TdP aVR, augmented vector right; aVL, augmented vector left; aVF, augmented vector foot; ECG, electrocardiogram

He was admitted to the medicine ward on continuous telemetry monitoring with intravenous/oral hydration, intravenous magnesium sulfate, and intravenous potassium chloride in an attempt to correct the electrolyte disturbances. While under monitoring, the patient suffered an episode of acute loss of consciousness. Telemetry recorded multiple episodes of PVCs with intermittent episodes of polymorphic ventricular tachycardia (VT) with a max heart rate of >200 bpm consistent with TdP (Figure 2). The loss of consciousness was witnessed during the rapid response team intervention with a duration of approximately 20 seconds. He was managed with an immediate bolus of magnesium sulfate and transferred to the coronary care unit for a higher level of care, cardiac monitoring, and continuous hemodynamic monitoring. Despite aggressive magnesium and potassium intravenous/oral replacements, the patient continued with persistent potassium depletion. Secondary causes for hypertension were explored, and workup for hyperaldosteronism was ordered (Table 1). Urine electrolyte quantification suggested renal potassium wasting. Plasma renin activity and plasma aldosterone concentration corresponded to decreased renin activity and aldosterone suppression. Plasma aldosterone concentration to plasma renin activity was 4.1, which goes against primary hyperaldosteronism. Careful history taking revealed the use of excessive licorice-containing supplements over the previous two months. The patient referred the excessive use of licorice supplements because a friend explained that the daily ingestion of large amounts of licorice can reduce potassium levels. The patient had hyperkalemia secondary to renal disease, and his primary care provider had prescribed sodium polystyrene. However, the patient was noncompliant with therapy due to diarrhea and was instead self-treating with natural supplements that would decrease his potassium levels.

Supportive therapy was provided. Telemetry and electrocardiogram monitoring showed progressive correction of the QT interval up to the baseline value. Hypokalemia slowly improved over the next seven days with daily requirements reaching as high as >120 mEq/L/day. The clinical course was favorable, and the patient was discharged after eight days with complete correction of the electrolyte derangements, correction of electrocardiographic changes, and clear advice to completely avoid the offending agent (licorice). The patient has remained stable and has had no further episodes of hypokalemia on outpatient follow-up.

## Discussion

Aldosterone is produced In the zona glomerulosa, and cortisol is produced in the zona fasciculata of the adrenal cortex. By stimulating mineralocorticoid receptors (MR) in renal tissues, they regulate blood pressure, electrolyte balance, and water balance [6]. Cortisol and aldosterone bind the mineralocorticoid receptors (MR) with similar affinity, but cortisol levels are 100-1000 times higher than that of aldosterone [7]. MR activation is regulated largely by the renin-aldosterone system thanks to the presence of the enzyme 11 beta-hydroxysteroid dehydrogenase type 2 (11HSD2), which inactivates cortisol to cortisone [7]. Cortisone is unable to stimulate MR, ensuring that the activation of the MR is mostly by aldosterone [7]. However, if the enzyme is blocked (as during prolonged intake of licorice), cortisol is able to bind and activate MR, which leads to pseudohyperaldosteronism [7].

Pseudohyperaldosteronism is a clinical condition characterized by sodium retention, hypertension, metabolic alkalosis, potassium wasting, and suppression of plasma renin and aldosterone levels [2]. The main active constituent of licorice is the prodrug glycyrrhizin, which is converted to 3β-monoglucuronyl-18β-glycyrrhetinic acid (3MGA) and 18β-glycyrrhetinic acid (GA) by intestinal bacteria possessing specialized β-glucuronidases [7]. Both 3MGA and GA are known to mimic an apparent mineralocorticoid excess through various mechanisms, including direct binding of the mineralocorticoid receptor in the kidney distal tubules and inhibiting 11β-hydroxysteroid dehydrogenase type 2, resulting in increased active cortisol concentration in the renal tissue [7]. This leads to mineralocorticoid receptor activation by cortisol via the inhibition of the enzymes necessary for its catabolism. This in turn suppresses the renin-angiotensin system, leading to a reduction in aldosterone and renin such as in our patient.

Clinically relevant effects can become evident with daily confectionery licorice consumption of >100 g over 1-4 weeks [8]. Our patient ingested licorice-containing products for more than two months in an attempt to reduce potassium levels with natural remedies. The patient had hyperkalemia secondary to renal disease, and his primary care provider had prescribed sodium polystyrene. However, the patient was noncompliant with therapy due to diarrhea and was instead self-treating with natural supplements that would decrease his potassium levels. The resulting pseudohyperaldosteronism secondary to licorice intoxication caused hypokalemia and TdP that could have brought about the patient’s demise. The chewy ropes of candy licorice twists are a common source of licorice and hence the metaphoric title of “Close Call From a Sweet Twist,” referring to the licorice-provoked TdP (Figure 2). This is another example of how poorly regulated food products may prove unsafe for patients.

Hypokalemia in this patient promoted significant prolongation of the QT interval and TdP. Syncope in this patient was attributed to polymorphic VT (TdP). Admission and monitoring during drug withdrawal are usually suggested for patients with markedly prolonged corrected QT (QTc) (>500 ms) or an increase in QTc of at least 60 ms compared with the predrug baseline value [5]. For patients with a single episode of TdP, treatment with intravenous magnesium and the correction of metabolic and electrolyte derangements and the removal of any inciting agent may be sufficient [5]. Intravenous magnesium is the agent of choice for immediate treatment of TdP irrespective of the serum magnesium level. The recommended dose is a 2 g intravenous bolus of magnesium sulfate followed by intravenous infusion at 3-20 mg/minute [5]. Temporary transvenous overdrive pacing is usually reserved for patients with long QT-related TdP who do not respond to intravenous magnesium [5]. For patients with a pre-existing permanent pacemaker, device reprogramming to increase the pacing rate can be beneficial. For patients with a pre-existing implantable cardioverter-defibrillator (ICD), the device can be reprogrammed to a prolonged detection time, delaying ICD shock for episodes of TdP that may self-terminate [5]. Pacing at rates of approximately 100 bpm will decrease the dispersion of refractoriness and the development of early afterdepolarizations, which may shorten the surface QT interval. Isoproterenol can also increase the sinus rate and decrease the QT interval as a temporizing measure before pacing [5].

Our patient was managed via the correction of metabolic derangements with aggressive intravenous potassium/magnesium replacements and the withdrawal of the offending agent. Shock therapy or pacing was not needed. Despite aggressive intravenous potassium and magnesium replacements, this patient continued with high daily requirements of potassium for seven days, some days exceeding 120 mEq. In volume-depleted patients, sodium and water delivery to the distal potassium secretory site may be substantially reduced. In this setting, the urine potassium concentration may be relatively high due to secondary hyperaldosteronism, but the urine volume and absolute amount of potassium excreted are relatively low. A potassium/creatinine ratio greater than 20 mEq/g has been suggested to indicate the presence of renal potassium wasting [9]. Our patient’s urine electrolyte quantification resulted in a potassium/creatinine ratio of 22.2 mEq/g, suggesting renal potassium wasting. Plasma renin activity was 0.24 ng/mL/hour (normal: 0.25-5.82 ng/mL/hour), and plasma aldosterone concentration was <1 ng/dL (normal: 3-16 ng/dL), which corresponds with renin and aldosterone suppression. The initial screening test is plasma renin activity to plasma aldosterone concentration, and most authors recommend the ratio to be 20-40 as a positive result for primary hyperaldosteronism [10]. Our patient’s plasma aldosterone concentration/plasma renin concentration ratio was 4.1, which goes against primary hyperaldosteronism and is in favor of pseudohyperaldosteronism.

## Conclusions

We herein report a life-threatening adverse effect of licorice on the cardiovascular system. Licorice is a commonly used natural product that is available in many forms. It is sometimes used as a natural supplement and sweetener that can be widely found in many food products. Food products are poorly regulated around the world, and in this case, this almost always results in the patient’s demise. Excessive ingestion can lead to an apparent mineralocorticoid excess through mechanisms involving glycyrrhizic acid. It may cause a cortisol-induced mineralocorticoid reaction, and it also has the capacity to directly stimulate the MR leading to a reduction in plasma potassium, sodium retention, metabolic acidosis, and hypertension. Hypokalemia can be severe in some patients and lead to fatal cardiac arrhythmias such as ventricular tachycardia and torsades de pointes. A careful history is essential in cases of refractive hypokalemia and renal potassium wasting, especially in elderly patients with underlying renovascular disease. Primary healthcare providers should be aware of the threatening adverse effects of licorice, treatment options, and diagnostic clues for pseudohyperaldosteronism.
